# Use of anti-tuberculosis drugs among newly diagnosed pulmonary tuberculosis inpatients in China: a retrospective study

**DOI:** 10.1186/s40249-016-0098-9

**Published:** 2016-01-21

**Authors:** Fei Huang, Hui Zhang, Qing Lv, Kaori D. Sato, Yan Qu, Shitong Huan, Jun Cheng, Fei Zhao, Lixia Wang

**Affiliations:** National Center for TB control and prevention, No 155 Changbai Road, Changping District Beijing, 102206 China; Duke Global Health Institute, Durham, NC USA; Bill & Melinda Gates Foundation, Beijing office, Beijing, China

**Keywords:** Tuberculosis, Inpatient, Drug, Regimen, Rationality

## Abstract

**Background:**

China’s national tuberculosis control program (NTP) provides free, first-line anti-tuberculosis (TB) drugs to pulmonary TB patients. This treatment regimen follows the World Health Organization’s (WHO) guideline. The objective of this paper is to evaluate the current status of anti-TB drug use for newly diagnosed pulmonary TB inpatients treated in prefecture- and county-level designated hospitals.

**Methods:**

Three prefecture-level hospitals and nine county-level hospitals were selected for the study. All newly diagnosed pulmonary TB inpatient medical records from 2012 were reviewed and doubly examined by two national senior physicians. The rational use of anti-TB drugs was evaluated based on criteria in line with WHO’s guideline.

**Results:**

Of the 2,060 total treatment regimens for TB, 53.1 % were found to be rational (1093/2060). The percentages in prefecture-level and county-level hospitals were 50.3 % (761/1513) and 60.7 % (332/547), respectively. The difference between the two levels of hospitals was statistically significant (Chi-square value = 17.44, *P* < 0.01). The percentages of rational treatment regimens for first-time hospitalizations and for two or more hospitalizations were 59.5 % (983/1653) and 27.0 % (110/407), respectively, with a statistically significant difference (Chi-square value = 138.00, *P* < 0.01). The overall use of second-line drugs (SLD) was 54.9 % (1131/2060). The percentages for prefecture-level and county-level hospitals were 50.6 % (766/1513) and 66.7 % (365/547), respectively. A statistically significant difference was found (Chi-square value = 42.06, *P* < 0.01). The use of SLD for inpatients hospitalized once and inpatients hospitalized twice or more was 58.4 % (966/1653) and 40.5 % (165/407), respectively, with a statistically significant difference (Chi-square value = 42.26, *P* < 0.01).

**Conclusions:**

Half of inpatients might be treated with irrational regimens, and the use of SLD was more appropriately dispensed in city-level hospitals than in county-level hospitals. Trainings and guidelines for health personnel, supervision led by health authorities and increased investment to designated hospitals may help to improve the rational use of anti-TB drugs.

**Electronic supplementary material:**

The online version of this article (doi:10.1186/s40249-016-0098-9) contains supplementary material, which is available to authorized users.

## Multilingual abstract

Please see Additional file 1 for translations of the abstract into the six official working languages of the United Nations.

## Background

According to the World Health Organization (WHO) estimate, China has one of the largest numbers of tuberculosis (TB) patients with multi-drug resistant tuberculosis (MDR-TB) in the world [1]. A major contributing factor to the rise of MDR-TB is non-adherence to or irrational TB treatment regimens [2]. Tuberculosis is not only a major public health issue in China, but is a social problem as well. With the second highest TB burden in the world, China accounted for 16 % of all TB cases that were notified in 2012 [1]. In the past two decades, China has made good progress in TB control via a vertical Center of Disease Control and Prevention (CDC) system. It has also played an important role in global TB control through the implementation of the Directly Observed Treatment, Short-course (DOTS) strategy and through strengthening TB case treatment and management, as evidenced by the national prevalence survey results conducted in 1990 and 2010, which confirmed that China achieved a 65 % decline in the prevalence of smear-positive TB [3, 4]. However, China is still facing a great challenge in advancing the reduction of TB morbidity and mortality and in effectively controlling MDR-TB. This challenge is further complicated by the current transition of responsibilities for TB care and control from the CDC to designated hospitals [5], so it is critical to understand the current status of rational TB drug use in hospitals. The integrated model, where designated hospitals, often functioning as general hospitals, provide TB care, aims to produce the best outcomes of TB care and treatment. Studies showed that designated hospitals had the lowest rate of unnecessary hospitalizations and placed the lowest financial burden on uncomplicated TB patients [6].

At present, the national tuberculosis control program (NTP) in China provides free, first-line anti-TB drugs (FLD). All TB drug dispensaries, including designated hospitals, are required to provide standard treatment regimens for newly diagnosed TB patients. However, the irrational use of anti-TB drugs is still a common occurrence in China and globally [7, 8], which may lead to serious implications for the quality and cost of services [9, 10]. In addition, irrationally used anti-TB drugs may result in resistance to TB and cause further transmission throughout the community [11]. There are several reasons why irrational use of anti-TB drugs has occurred: ambiguity in clinical guidelines, insufficient diagnostic modalities, and poor documentation concerning referred patients, etc. [12].

Although there are published studies describing anti-TB drug use in China [13–18], they mainly focus on either the use of FLD or second-line anti-TB drugs (SLD), independently. To determine the situation of anti-TB drug use in China, including both FLD and SLD, we conducted a retrospective study in the China Ministry of Health (MOH)-Gates Foundation TB project pilot cities, which included Zhengjiang City, Jiangsu Province in the east, Yichang City, Hubei Province in central China, and Hanzhong City, Shaanxi Province in the west, and selected TB inpatients as a sample in order to acquire reliable data and information.

## Methods

### Study design

The goal of the China MOH-Gates Foundation TB project was to develop comprehensive models for TB control and evaluate their feasibility and effectiveness. To better design the intervention measures and collect baseline data for future impact assessments, a baseline survey, consisting of anti-TB drug use, among other questions, was conducted.

The three prefecture-level hospitals in Zhenjiang, Yichang and Hanzhong were all upper second-class hospitals that specialized in infectious diseases. These hospitals were designated by local health authorities to diagnose and treat MDR-TB patients living in each respective city and nearby counties before 2010. Three county-level designated hospitals were randomly selected from each city based on GDP per capita (i.e., low, middle and high levels), and in total nine county-level hospitals were surveyed. Seven of the nine county-level hospitals were upper second-class general hospitals and were designated for the diagnosis and treatment of TB patients before 2013. The other two hospitals were middle second-class general hospitals and were designated only for the diagnosis and treatment of TB inpatients, while the local CDC was responsible for the diagnosis and treatment of TB outpatients.

### Data sources and collection

Information on TB inpatients were stored via medical records in all hospitals, including diagnosis results, treatment regimens, use of auxiliary drugs and clinical examinations. In total, 4,076 medical records with a discharge diagnosis of pulmonary TB and a discharge date in 2012 were imported from the hospital information system, and 482 records were excluded due to drug-resistance, HIV-positive status and/or obsolete TB. The other The remaining 3,594 pulmonary TB inpatient medical records were then examined independently by two chest physicians. First, one physician recorded treatment regimens prescribed for and used by each patient during their hospitalization, the dates of hospital admission and discharge, and personal information (e.g., sex, age, etc.) using a designated form. According to the study design, we did not collect information on the use of auxiliary drugs and clinical examinations. Afterwards, the second physician checked the records again to ensure that the information was recorded correctly. Inpatients who were treated for other diseases were excluded from the study, as those with co-morbidities often did not begin TB treatment until other health problems were controlled or until they received complex treatments to address co-morbidities.

While examining the medical records, in-depth interviews with the deputy deans of the hospitals and focus group interviews with TB physicians were conducted by trained interviewers to understand the behaviors of the deputy deans and TB physicians.

### Data management and analysis

In China, all diagnosed pulmonary TB cases are required to be reported and recorded in the national electronic TB Information Management System (TBIMS), including information regarding demographics, diagnosis date and results, treatment history, etc. We acquired the patients’ information from the TBIMS and linked it with the hospitalization medical record database. We only included new TB patients (i.e., those never treated for TB), and excluded retreatment cases, most of whom were chronic TB patients. Finally, 1,653 cases with 2,060 medical records were analyzed.

The rational use of treatment regimens was then analyzed and assessed based on treatment guidelines for the initial phase (i.e., the first two months) and the continuation phase (i.e., the last four months). The rationality of treatment regimens was determined by how strictly treatments followed the WHO guidelines, which was 2HRZE/4HR for new TB patients [19]. Deviations from the WHO guidelines were discussed with a third chest physician from the study hospital to analyze the reasons for modifying the inpatient’s TB treatment regimen. 2HRE/4HR was regarded as rational if the TB inpatients were over 65 years old, and 2HRZES/6HRE and 3HRZE/6HRE were rational if the TB inpatients were only resistant to isoniazid. In the case of side effects, 2HRZE/4HR was rational if rifampicin was replaced with rifapentine and/or if pyrazinamide was replaced with kanamycin, levofloxacin or other SLDs.

### Ethics Review

The study protocol was approved by the China CDC institutional review board, as the study only used routine data and did not involve any human participation. Therefore, the application for waiving the informed consent was accepted by the institutional review board.

## Results

### Demographics of the study population

There were 1,653 TB inpatients included in the study, of which 70.7 % were male and 29.3 % female. Out of the five age groups, TB cases in the 60 years old and over group accounted for the largest percentage at 33.6 %, while the 0–14 age group accounted for the smallest percentage (0.7 %). In general, there was a negative relationship between the proportion of inpatients and age group (Table 1).

**Table 1:** TB inpatients by age and sex

Age group	Male		Female		Total	
	n	%	n	%	n	%
0-	7	0.6	5	1.0	12	0.7
15-	257	22.0	126	26.0	383	23.2
30-	191	16.3	93	19.2	284	17.2
45-	306	26.2	113	23.3	419	25.3
60-	408	34.9	147	30.4	555	33.6
Total	1169	100.0	484	100.0	1653	100.0

### Level of hospital and number of hospital admissions

Among all 1,653 TB inpatients, 1,150 cases (69.6 %) were from prefecture-level hospitals, while 503 cases (30.4 %) were from county-level hospitals. We found that 85.0 % were hospitalized only once. At the prefecture-level hospitals, 81.5 % of inpatient TB cases were only hospitalized once, while the proportion was 93.5 % at the county-level hospitals. A statistically significant difference was found between the two types of hospitals (Chi-square value = 36.69, *P* < 0.01) (Table 2).

**Table 2:** TB inpatients by hospital level and hospital admission

Hospital level	One hospital admission		Hospital admissions >1 time		Total
	n	%	n	%	
Prefecture	937	81.5	213	18.5	1150
County	468	93.0	35	7.0	503
Total	1405	85.0	248	15.0	1653

### Rational use of treatment regimens

The overall rate of rational use of treatment regimens was 53.1 %. The percentages in prefecture-level and county-level hospitals were 50.3 % and 60.7 %, respectively, with a statistically significant difference (Chi-square value = 17.44, *P* < 0.01). The rational use of treatment regimens for first-time hospitalizations and for two or more hospital admissions was 59.5 % and 27.0 %, respectively. The difference was statistically significant (Chi-square value = 138.00, *P* < 0.01), but the interpretation was not clear. Prefecture-level (Chi-square value = 118.95, *P* < 0.01) and county-level hospitals showed similar results (Chi-square value = 7.85, *P* < 0.01) (Table 3).

**Table 3:** Rational use of treatment regimens by hospital level and hospital admission

Hospital level	No of Hospital admission	Rational		Irrational		Total
		N	%	N	%	
Prefecture	1^st^	669	58.2 %	481	41.8 %	1150
	2^nd^ − +	92	25.3 %	271	74.7 %	363
	Sub-total	761	50.3 %	752	49.7 %	1513
County	1^st^	314	62.4 %	189	37.6 %	503
	2^nd^-	18	40.9 %	26	59.1 %	44
	Sub-total	332	60.7 %	215	39.3 %	547
Total	1^st^	983	59.5 %	670	40.5 %	1653
	2^nd^-	110	27.0 %	297	73.0 %	407
	Sub-total	1093	53.1 %	967	46.9 %	2060

### Use of SLD

The overall use of SLD for all study cases was 54.9 %. The percentages in prefecture- and county-level hospitals was 50.6 % and 66.7 %, respectively, with a significant difference (Chi-square value = 42.06, *P* < 0.01). The use of SLD for inpatients hospitalized once and inpatients hospitalized twice or more was 58.4 % and 40.5 %, respectively, with a significant difference (Chi-square value = 42.26, *P* < 0.01). The prefecture-level hospitals showed similar results; however, (Chi-square value = 30.39, *P* < 0.01) the county-level hospitals had a significantly higher rate of SLD use (Chi-square value = 0.62, *P* = 0.43) (Table 4).

**Table 4:** Use of SLD by hospital level and hospital admission time

Hospital level	Hospital admission	Use of SLD		Nonuse of SLD		Total
		n	%	n	%	
Prefecture	1^st^	628	54.6 %	522	45.4 %	1150
	2^nd^-	138	38.0 %	225	62.0 %	363
	Sub-total	766	50.6 %	747	49.4 %	1513
County	1^st^	338	67.2 %	165	32.8 %	503
	2^nd^-	27	61.4 %	17	38.6 %	44
	Sub-total	365	66.7 %	182	33.3 %	547
Total	1^st^	966	58.4 %	687	41.6 %	1653
	2^nd^-	165	40.5 %	242	59.5 %	407
	Sub-total	1131	54.9 %	929	45.1 %	2060

### Treatment regimen and SLD use

The use of SLD for inpatients treated with rational and irrational regimens was 49.6 % and 50.4 %, respectively, with a significant difference (Chi-square value = 12.03, *P* < 0.01). Similar trends were observed at prefecture- (Chi-square value = 50.76, *P* < 0.01) and county-level hospitals (Chi-square value = 19.00, *P* < 0.01) (Table 5).

**Table 5:** Use of SLD by treatment regimen

Hospital level	Use of SLD	Rational regimen		Irrational regimen		Total
		N	%	N	%	
Prefecture	Yes	316	41.3 %	450	58.7 %	766
	No	445	59.6 %	302	40.4 %	747
	Sub-total	761	50.3 %	752	49.7 %	1513
County	Yes	245	67.1 %	120	32.9 %	365
	No	87	47.8 %	95	52.2 %	182
	Sub-total	332	60.7 %	215	39.3 %	547
Total	Yes	561	49.6 %	570	50.4 %	1131
	No	532	57.3 %	397	42.7 %	929
	Sub-total	1093	53.1 %	967	46.9 %	2060

## Discussion

This study showed that about half of the TB inpatients were treated with irrational regimens and that half of them were also treated with SLD. The treatment regimens given to TB inpatients for first-time hospital admissions were more rational than for those who were hospitalized two or more times. This finding was evident in both the prefecture- and county-level hospitals. Furthermore, 15 % of TB inpatients were hospitalized twice or more, and the percentage was higher in prefecture hospitals than in county hospitals, which was not surprising as the prefecture hospitals received more complex and severe cases referred by the county hospitals.

Many factors may be associated with the irrational use of TB treatment regimens and the overuse of SLD, especially in the county hospitals. First, the high deviation from standard guidelines may be due to revenue generation. Perverse financial incentives given to hospitals have likely been a main factor affecting the irrational use of medicines in China [13, 20, 21]. Chinese hospitals are not fully financed by public funds; thus, they need to generate a substantial amount of revenue to cover their operational costs. In this study, the government’s direct investment to the prefecture hospitals in 2012 accounted for about 12.8 % of total revenue, while the proportion in the county hospitals was about 3.2 %. In addition, the income levels of doctors and other health professionals in Chinese hospitals are closely associated with the degree of revenue generation. Chinese physician salaries are often very low, supplemented by so-called bonus payments that are linked to revenue generation. In order to increase the level of revenue for hospitals and bonus payments, physicians often admit more patients, overuse unnecessary tests, and overprescribe medications, particularly expensive ones [22] since first-line anti-TB drugs are paid for by government earmarked funds. Thus, hospitals do not obtain any financial benefits from the use of FLD. Therefore, it is not surprising to see that doctors prefer to prescribe SLD, which can bring revenue to the hospital and increase bonus payments for themselves.

Second, quality assurance systems have not been well implemented in most Chinese county general hospitals, and it is difficult to supervise the doctors’ behaviors [23]. Although the CDC is still responsible for TB control and prevention in China, the tasks of patient diagnosis and treatment have now been shifted from the CDC to the designated hospitals. The CDC, particularly at the county level, lacks the authority and/or capacity to monitor and supervise the TB services provided by the hospitals. They are often in a weak position to point out the irrationality of treatment regimens prescribed by the hospitals, as most of the CDC public health doctors are not licensed physicians [24].

The SLD was more appropriately used in prefecture-level hospitals than in county-level hospitals. A possible reason for this is that physicians in the prefecture hospitals are typically better trained than those in the county hospital. The three prefecture-level hospitals were TB designated hospitals and were also designated for MDR-TB diagnosis and treatment since 2010. They served as reference centers for TB diagnosis and treatment for their respective regions. At least one clinician from the TB department at the prefecture hospitals received training provided by the China CDC national experts. However, with the exception of one county hospital that was designated for TB care in 2002, the other county hospitals were designated for TB care between 2011 and 2012, and were not offered training opportunities over the past years. Instead, they practiced TB services based on the TB treatment protocols provided in the national guidelines. Due to this existing situation, the doctors from prefecture hospitals may have more knowledge about TB treatment regimens and practice than those from county hospitals. This may explain why the rationality of FLD was higher in the prefecture hospitals than in the county hospitals, while the use of SLD was lower in the prefecture hospitals than in the county hospitals. Many doctors from the county hospitals did not receive any training on TB treatments and did not know how to manage complex cases.

Our study investigated different types of hospitals at the prefecture and county levels. The medical records were doubly examined by two experienced physicians. In addition, we linked the data from the medical records to the routine surveillance system to assess the rationale of treatment in different phases for each inpatient. The results reveal existing problems in TB care that may have occurred while shifting from CDC model to hospital model, and should be addressed and solved as soon as possible. However, there were three limitations to the study due to time and resource constraints. Firstly, we did not examine the rationality of TB outpatient treatment regimens. Secondly, we did not analyze the cost of hospitalization, as irrational treatment regimens may result in high cost of hospitalization. Thirdly, we did not evaluate the rationality of treatment regimens for TB outpatients, nor the use of auxiliary drugs and clinical examinations, which also play a very important role in treatment outcome. We will try to cover these limitations in our next study in the three project sites.

## Conclusion

The irrational use of anti-TB drugs is a common global problem, and it is often neglected even though there are many pressing concerns. The impact of irrational anti-TB drug use could lead to serious implications, including the development of MDR-TB [11]. Premature introduction of SLD for general TB patients may induce more drug-resistance, which in turn can result in the lack of effective drugs when general TB patients develop MDR-TB [9]. At the same time, there is a risk that new drugs will be used in inappropriate treatment regimens, introducing the subsequent risk of resistance development to these new drugs. Therefore, the rational use of anti-TB drugs is critically important for all TB dispensaries. To improve rational anti-TB drug use, the National Drug Policy should first be concerned with not only the supply of safe, effective and appropriate drugs but also with the way in which these drugs are prescribed and dispensed by health personnel, which means TB doctors should be well-trained on how to treat TB patients appropriately. Secondly, the health administrative department should develop regulations and establish supervisory groups to oversee doctors’ behaviors. Thirdly, the Chinese government should increase direct investments to the designated hospitals to avoid profit-seeking behavior. Government funding and supervision are key factors for the successful operation of designated TB hospitals [23, 25]. In addition, doctors’ incomes should not be incentivized by prescribing irrational drugs. Rather, the funding and focus should be placed on following appropriate treatment regimens and should be related to health outcomes. Last but not least, the national TB control program should update the guidelines and hold training courses to instruct and guide doctors on proper TB drug regimens.

## Additional file

Additional file 1:
**Multilingual abstracts in the six official working languages of the United Nations.** (PDF 371 kb)
