# Supplementary material for: Use of anti-tuberculosis drugs among newly diagnosed pulmonary tuberculosis inpatients in China: a retrospective study
Source: Infect Dis Poverty. 2016 Jan 21;5:2. doi: 10.1186/s40249-016-0098-9 (PMC4720996; doi:10.1186/s40249-016-0098-9)
Supplement: Additional file 1: — Multilingual abstracts in the six official working languages of the United Nations. (PDF 371 kb) [file 40249_2016_98_MOESM1_ESM.pdf]

Translation of the abstract into the six official working languages of the United Nations

استخدام الأدوية المضادة للسل بين مرضى المنامات المصابين السل الرئوي الذين تم تشخيصهم حديثًا في الصين: دراسة استعادية

Fei Huang, Hui Zhang, Qing Lv, Kaori D. Sato, Yan Qu, Shitong Huan, Jun Cheng, Fei Zhao, Lixia Wang

ملخص

الخلفية: يقدم البرنامج الوطني الصيني لمكافحة السل (NTP) الخيار الأول من الأدوية المضادة للسل (TB) لمرضى السل الرئوي. يتبع هذا النظام العلاجي توجيهات منظمة الصحة العالمية (WHO). وتهدف هذه الورقة إلى تقييم الوضع الحالي لاستخدام الأدوية المضادة لمرض السل من قبل مرضى المنامات والذيم تم تشخيص إصابتهم بمرض السل الرئوي مؤخرًا والذين تم علاجهم في مستشفيات محددة على مستوى المقاطعات أو على مستوى الدولة.

الوسائل: تم اختيار ثلاث مستشفيات على مستوى المقاطعة وتسع مستشفيات على مستوى الدولة لأغراض هذه الدراسة. تمت مراجعة جميع السجلات الطبية منذ عام 2012 والخاصة بمرضى المنامات المصابين بمرض السل الرئوي، وتدقيقها مرتين من قبل أطباء مقيمين ذوي خبرة. تم تقييم الاستخدام الرشيد للأدوية المضادة للسل بناء على معايير تتماشى مع توجيهات منظمة الصحة العالمية.

النتائج: من بين 2060 نظامًا علاجيًا لمرض السل، وُجد أن %53.1 منها رشيدة (2060/1093). وكانت النسب في المستشفيات على مستوى المقاطعات ومستوى الدولة %50.3 (1513/761) و %60.7 (574/332) على التوالي. كما كان الفرق بين أنواع المستشفيات حسب مستوياتها كبير من الناحية الإحصائية (قيمة مربع كاي=17.44، 0,01>P). وكانت النسب المئوية لأنظمة العلاج الرشيدة لمن يدخَلون إلى المستشفى لأول مرة %59.5 (1653/983) ولمن يدخَلون إلى المستشفى للمرة الثانية أو أكثر %27.0 (407/110)، مع وجود فرق كبير من الناحية الإحصائية (قيمة مربع كاي= 138.00، 0,01>P). وبلغ الاستخدام العام لأدوية الخيار الثاني (SLD) %54.9 (2060/1131). أما النسب المئوية للمستشفيات على مستوى المقاطعات وعلى مستوى الدولة فقد بلغت %50.6 (1513/766) و %66.7 (547/365) على التوالي. ووُجد فرق كبير ذو دلالة إحصائية (قيمة مربع كاي=42.06، 0,01>P). وبلغت نسب استخدام أدوية الخيار الثاني للمرضى المدخَلين إلى المستشفى لأول مرة وغيرهم المدخَلين للمرة الثانية أو أكثر %58.4 (1653/966) و %40.5 (407/165) على التوالي، مع فرق كبير ذو دلالة إحصائية (قيمة مربع كاي=42.26، 0,01>P).

الاستنتاجات: من المحتمل أن يكون قد تم علاج نصف المرضى المقيمين باستخدام أنظمة علاجية غير رشيدة. كان صرف أدوية الخيار الثاني أكثر ملائمة في المستشفيات على مستوى المدينة منه في المستشفيات على مستوى الدولة. قد تساعد المبادئ التوجيهية والدورات التدريبية لموظفي الصحة، والتي تشرف عليها السلطات الصحية، إلى جانب زيادة الاستثمار في مستشفيات محددة في تحسين الاستخدام الرشيد للأدوية المضادة للسل.

Translated from English version into Arabic by Shada Salameh, through

TRANSLATORS WITHOUT BORDERS

中国初治住院肺结核患者抗结核药物使用：一项回顾性研究

黄飞，张慧，吕青, Kaori D. Sato，屈燕，桓世彤，成君，赵飞，王黎霞

摘要

背景：中国结核病防治规划为肺结核患者提供免费的一线抗结核药物，并按照世界卫生组织的指南制定化疗方案。本文目的是评价初治肺结核患者在地市级和县区级定点医院治疗时抗结核药物的使用情况。

方法：本研究共选择 3 个地市级定点医院和 9 个县区级定点医院。由二个国家级资深专家对所有初治肺结

核患者在 2012 年的住院病案进行核查，并依据世界卫生组织指南的标准来评价抗结核药物的合理使用。
结果：2060 例化疗方案中合理的比例为 53.1%（1093/2060）。地市级和县区定点医院的合理比例分别为 50.3%（761/1513）和 60.7%（332/547）,并有显著性差异（卡方值=17.44, P<0.01）。首次住院和第二次及以上住院化疗方案合理的比例分别为 59.5%（983/1653）和 27.0%（110/407），并有显著性差异（卡方值=138.00, P<0.01）。二线药物合理使用的比例为 54.9%（1131/2060，在地市级和县区级医院分别为 50.6%（766/1513）和 66.7%（365/547），并有显著性差异（卡方值=42.06, P<0.01）。首次住院和第二次及以上住院的二线药物合理使用的比例分别为 58.4%（966/1653）和 40.5%（165/407），并有显著性差异（卡方值=42.26, P<0.01）。
结论：50%住院患者的化疗方案不合理，县区级定点医院二线抗结核药物合理使用的比例高于地市级定点医院。对医务人员进行培训和指导、卫生行政部门加强监管和加大对定点医院的投入可能有助于提高抗结核药物的使用。

Translated from English version into Chinese by Huang Fei, through

Utilisation de médicaments antituberculeux pour des patients hospitalisés atteints de tuberculose pulmonaire récemment diagnostiquée en Chine : étude rétrospective

Fei Huang, Hui Zhang, Qing Lu, Kaori D. Sato, Yan Qu, Shitong Huan, Jun Cheng, Fei Zhao, Lixia Wang

Résumé

Contexte : Le programme national chinois de lutte contre la tuberculose prévoit la fourniture gratuite de médicaments antituberculeux de première ligne aux patients atteints de tuberculose pulmonaire. Ce schéma de traitement suit la ligne directrice de l'Organisation mondiale de la Santé (OMS). Le présent article fait un état des lieux de l'utilisation de médicaments antituberculeux pour des patients atteints de tuberculose pulmonaire récemment diagnostiquée hospitalisés dans des hôpitaux de préfecture et de circonscription désignés.

Méthodes : Trois hôpitaux de préfecture et neuf hôpitaux de circonscription ont été sélectionnés pour l'étude. Les dossiers de tous les patients hospitalisés atteints de tuberculose pulmonaire récemment diagnostiquée depuis 2012 ont été revus et examinés par deux médecins de haut niveau national. L'utilisation rationnelle des antituberculeux a été évaluée sur la base de critères conformes à la ligne directrice de l'OMS.

Résultats : Sur les 2060 traitements antituberculeux, 53,1 % ont été jugés rationnels (1093/2060). Les pourcentages dans les hôpitaux de préfecture et de circonscription étaient respectivement de 50,3 % (761/1513) et 60,7 % (332/547). La différence entre les deux échelons d'hôpitaux était statistiquement significative (valeur de chi-deux = 17,44, P < 0,01). Les pourcentages de traitements rationnels pour les premières hospitalisations et pour deux hospitalisations ou plus étaient respectivement de 59,5 % (983/1653) et 27,0 % (110/407), avec une différence statistiquement significative (valeur de chi-deux = 138,00, P < 0,01). Des médicaments de deuxième ligne ont été utilisés dans 54,9 % du total des cas (1131/2060). Les pourcentages dans les hôpitaux de préfecture et de circonscription étaient respectivement de 50,6 % (766/1513) et 66,7 % (365/547). La différence est statistiquement significative (valeur de chi-deux = 42,06, P < 0,01). Le taux d'utilisation de médicaments de deuxième ligne pour les patients hospitalisés une fois et ceux hospitalisés deux fois ou plus était respectivement de 58,4 % (966/1653) et 40,5 % (165/407), avec une différence statistiquement significative (valeur de chi-deux = 42,26, P < 0,01).

Conclusions : La moitié des patients hospitalisés ont peut-être été traités de façon irrationnelle et les médicaments de seconde ligne ont été utilisés de façon plus appropriée dans les hôpitaux des villes que dans les hôpitaux de circonscription. Des formations et des directives à l'intention du personnel de santé, une supervision par les

autorités de la santé et un investissement accru dans les hôpitaux désignés pourraient contribuer à améliorer l'usage rationnel des médicaments antituberculeux.

Translated from English version into French by Suzanne Assenat, through

TRANSLATORS
WITHOUT BORDERS

Использование противотуберкулезных препаратов у госпитализированных пациентов с впервые выявленным туберкулёзом лёгких в Китае Ретроспективное исследование

Фэй Хуан, Хуэй Чжан, Цин Лв, Каори Д. Сато, Янь Цюй, Шитонг Хуань, Жунь Чэнь, Фэй Чжао, Ликсия Ван

Реферат

История вопроса: Национальная программа по контролю за распространением туберкулеза (НПКТ) Китая предусматривает обеспечение больных туберкулезом легких бесплатными противотуберкулезными препаратами первого ряда. Этот курс лечения составлен в соответствии с рекомендациями Всемирной организации здравоохранения (ВОЗ). Целью данной статьи является оценка актуального состояния использования противотуберкулезных препаратов у впервые выявленных госпитализированных пациентов с туберкулезом легких, проходящих лечение в выделенных окружных больницах.

Методы: Для проведения исследования были отобраны три окружных и девять уездных больниц. Двумя главными врачами были изучены истории болезни всех впервые выявленных госпитализированных пациентов с туберкулезом легких с 2012 года. Было оценено рациональное использование противотуберкулезных препаратов на основе критериев, установленных в соответствии с рекомендациями ВОЗ.

Результаты: Из 2060 курсов лечения туберкулеза 53,1% были признаны рациональными (1093/2060). Процентные значения для больниц окружного и уездного значения составили 50,3% (761/1513) и 60,7% (332/547) соответственно. Выявлено статистически значимое расхождение между больницами двух уровней (значение хи-квадрат=17,44, P<0,01). Процент рациональных курсов лечения для первой госпитализации и для двух и более госпитализаций составил 59,5% (983/1653) и 27,0% (110/407) соответственно, со статистически значимым расхождением (значение хи-квадрат=138,00 P<0,01). Общее использование препаратов второй линии (SLD) составило 54,9% (1131/2060). Процентные значения для больниц окружного и уездного значения составили 50,6% (766/1513) и 66,7% (365/547) соответственно. Определено статистически значимое расхождение (значение хи-квадрат=42,06, P<0,01). Использование препаратов второй линии (SLD) для пациентов, госпитализированных один раз и госпитализированных дважды или более составил 58,4% (966/1653) и 40,5% (165/407) соответственно, со статистически значимым расхождением (значение хи-квадрат=42,26, P<0,01).

Выводы: Половина госпитализированных пациентов могла получить нерациональные курсы лечения, а использование препаратов второй линии было более адекватным в больницах городского значения, чем в больницах уездного значения. Рациональность использование противотуберкулезных препаратов может быть повышена путем подготовки и обучения медицинского персонала, наблюдением органов здравоохранения и повышением инвестиций в выделенные больницы.

Translated from English version into Russian by Alena Hrybouskaya, through

Uso de medicamentos contra la tuberculosis en pacientes hospitalizados a los que se les ha diagnosticado tuberculosis pulmonar en China: Un estudio retrospectivo

Fei Huang, Hui Zhang, Qing Lv, Kaori D. Sato, Yan Qu, Shitong Huan, Jun Cheng, Fei Zhao, Lixia Wang

Resumen

Contexto: El programa nacional de control de la tuberculosis de China (NTP, por sus siglas en inglés) ofrece medicamentos gratuitos, de primera línea contra la tuberculosis (TB) a pacientes con TB pulmonar. Este régimen de tratamiento sigue las directrices de la Organización Mundial de la Salud (OMS). El objetivo del presente documento es evaluar el estado actual del uso de medicamentos contra la tuberculosis para pacientes hospitalizados a quienes se les ha diagnosticado tuberculosis pulmonar, tratados en hospitales designados a nivel de prefectura o condado.

Métodos: Para este estudio se seleccionaron tres hospitales de prefectura y nueve hospitales de condado y nueve hospitales de condado. Dos médicos experimentados nacionales revisaron y volvieron a revisar una segunda vez todos los expedientes médicos de los pacientes hospitalizados con TB pulmonar disponibles desde 2012. El uso racional de medicamentos contra la tuberculosis se evaluó en base a criterios adaptados a las directrices de la OMS.

Resultados: De los 2 600 regímenes de tratamiento para la TB, el 53,1% resultó ser racional (1093/2060). Los porcentajes a nivel de prefectura y a nivel de condado fueron del 50,3% (761/1513) y del 60,7% (332/547), respectivamente. La diferencia entre los dos niveles de hospitales fue significativa en términos estadísticos (valor chicuadrado=17,44, $P<0,01$). El porcentaje de regímenes de tratamiento racional para hospitalizaciones por primera vez y para dos o más hospitalizaciones fue del 59,5% (983/1653) y del 27,0% (110/407), respectivamente, con una diferencia significativa en términos estadísticos (valor chicuadrado=138,00, $P<0,01$). El uso global de medicamentos de segunda línea (SLD) fue del 54,9% (1131/2060). Los porcentajes a nivel de prefectura y a nivel de condado fueron del 50,6% (766/1513) y del 66,7% (365/547), respectivamente. Se encontró una diferencia significativa en términos estadísticos (valor chicuadrado=42,06, $P<0,01$). El uso de SLD para pacientes hospitalizados una vez y para pacientes hospitalizados dos o más veces fue del 58,4% (966/1653) y del 40,5% (165/407), respectivamente, con una diferencia significativa en términos estadísticos (valor chicuadrado=42,26, $P<0,01$).

Conclusiones: Se podrá tratar a la mitad de los pacientes con regímenes irracionales, y el uso de SLD se suministró más adecuadamente en hospitales de ciudad que en hospitales de condado. La formación y directrices sobre salud personal, la supervisión dirigida por las autoridades sanitarias y una mayor inversión en hospitales designados puede ayudar a mejorar el uso racional de los medicamentos contra la tuberculosis.

Translated from English version into Spanish by NTRAD, through

TRANSLATORS
WITHOUT BORDERS
